# Perseverative Cognition and Snack Choice: An Online Pilot Investigation

**DOI:** 10.3390/bs11030033

**Published:** 2021-03-11

**Authors:** Timothy M. Eschle, Dane McCarrick

**Affiliations:** 1 Psychology Department, School of Education and Social Sciences, University of the West of Scotland, Paisley PA1 2BE, UK; 2 School of Psychology, Faculty of Medicine and Health, University of Leeds, Leeds LS2 9JT, UK; psdjm@leeds.ac.uk

**Keywords:** perseverative cognition, rumination, stress, food choice, obesity

## Abstract

Perseverative cognition (PC), consisting of worry and rumination, has been consistently linked to a variety of poorer health outcomes, namely via the worsening of stress-induced health risk behaviours. However, research into PC and unhealthy food choice, a key health behaviour, still remains relatively unexplored. In the current pilot investigation, 284 participants were recruited to take part in an online food choice paradigm before completing the Perseverative Thinking Questionnaire (PTQ) and the Brief State Rumination Inventory (BSRI). As a reduced availability of unhealthy snacks has been shown to improve snack choice, participants were randomly allocated to either an even condition (a 3:3 ratio of ≤99 kcal and ≥199 kcal snacks) or an uneven condition (a 4:2 ratio in favour of ≤99 kcal snacks). It was hypothesized that higher levels of PC may predict greater instances of poorer snack choices across, or even within, this paradigm. Despite an increase availability of lower calorie snacks leading to a healthier snack choice, both state and trait PC measures did not significantly influence snack choice irrespective of this varying availability. Although, marginal trends were found for higher state PC and higher calorie crisp selections. The current pilot therefore adds to the growing literature advocating for the use of behavioural economic tactics to engender healthier food choices, yet further work is needed to unpick the mediating role of PC (and its components) in snack consumption paradigms.

## 1. Introduction

Obesity presents a monumental challenge to public health. A significant cause of surplus weight gain and body mass index (BMI) is poor dietary choices, namely excessive energy intake and the substantial consumption of calorie dense and highly processed foods [1]. For decades, behavioural research has attempted to highlight the contribution of an individual’s food environment and the surrounding obesogenic cues that may function to direct dietary choices towards innutritious, calorie dense foods [2,3]. This is further supported by the successful application of a number behavioural economic tactics; whereby reducing the availability, accessibility, and associated cues to innutritious foods (or alternatively increasing these cues towards healthier foods), can lead to improved food choice [4,5]. Associated literature has also sought to test the efficacy of such behavioural approaches, even when monitoring for several cognitive and demographic predictors of food choice including, impulsivity, cognitive load, social norms and social economic status [6,7,8]. However, the precise role of other moderating mechanisms such as stress and the affective components of food choice have thus far been overlooked.

The ongoing COVID-19 pandemic restrictions, such as those imposed within the United Kingdom during 2020, further emphasize the role of mental health status in the modulation of health behaviours within an individual’s environment [9,10]. Indeed, the impact of psychological stress has long been implicated in a variety of poorer health outcomes [11,12,13], with stress-induced health behaviours having been found to stimulate or intensify these further [14]. For instance, high cortisol reactivity has been shown to result in increased snack intake [15], while individuals with elevated stress levels have been found to display a higher preference for energy rich foods high in fat and refined sugar [16,17,18,19] and a converse intake of vegetables and consumption of main meals [20]. All of which, have been found to correlate with changes to BMI and associated comorbidities [19,21,22]. Psychological stress, therefore, plays an important role in appetite regulation and preferences for energy intake [23].

Important advancements in stress theory have led to the widening of investigation in an attempt to understand the common response to psychological stress. Originally proposed by Brosschot et al. [24], the Perseverative Cognition (PC) Hypothesis posits itself as the underpinning link between stress factors and prolonged physiological activation, resulting in pathogenic states. PC itself is the process through which cognitive representations of past stressors assimilate (through rumination) and feared events in the future take hold (via worry). Research has shown that failure to disengage from this damaging cognitive loop can have detrimental effects on health when unconstrained over sustained periods of time [25,26]. Indeed, it is proposed that PC meditates the prolonging of the harm omitted from the original stress stimuli by the sustained activation of autonomic, cardiovascular and endocrine systems via worry [27,28]; interestingly, at times, over and above those of the daily hassles themselves [28]. While less documented, PC has also shown to lead to alterations in health behaviours themselves including sleep imbalances, alcohol consumption and, of interest here, poorer dietary habits [26]. For instance, higher levels of work-based rumination have been found to lead to the increased consumption of unhealthy foods (relative to low ruminators), potentially as a means to distract from these unpleasant cognitions or to cope with the relative emotional reaction to such thoughts [29].

However, despite the link between PC, stress, and nutritional preferences it is apparent further research is needed to explore this as a modulator of food choice within its own right. Given the small, but growing, literature outlined here, it is possible that higher levels of trait and state PC may lead to unhealthy food choice, even in the presence of an established behavioural economic intervention (such as increased availability of healthy snack options). Exploring such an assumption would not only have ramifications for its inclusion as a modulator on a theoretical level, but would also allow for future interventionists to explore the importance of longer term strategies (e.g., cognitive-behavioural) that seek to identify and modify the recurring negative thoughts that [30,31,32], in turn, mitigate against PC-induced health-risk behaviours. Therefore, the current pilot study seeks to explore the contribution of PC to snack choice in an environment where there is reduced availability of high calorie options relative to a setting in which presents an equal opportunity for healthier and unhealthy snacks. Given the established premise that stressed (or high worrying/ruminating) individuals will seek out high fat or sugar, energy dense snacks, the current study proposed to investigate the effects of PC, and the altering availability of healthier snacks within this framework, by focusing on crisps and chocolate bars that vary in calorie content. Although it is axiomatic that there is more to food choice than calorie content alone, it is a key factor in weight management [33] and an important factor in an individual’s food choice [34].

Therefore, it was predicted that higher state and trait levels of PC would lead to a preference for higher calorie snacks in both the crisp (Hypothesis 1a) and chocolate (Hypothesis 1b). Secondly, it was further hypothesized that participants exposed to an increased availability of lower calorie snacks (i.e., the 4:2 uneven condition) will be more likely to opt for the “healthier” (i.e., lower kcal) choice, relative to those participants who have an equal availability of both lower and higher calorie snacks (i.e., even condition) for both crisps (Hypothesis 2a) and chocolate bars (Hypothesis 2b). In all of these analyses, trait and state PC will be assessed separately, as although they are conceptually related, they are considered separate constructs that have distinct effects on the physiological correlates of the stress response [35,36]. Moreover, while other investigations have considered the various components of PC itself (e.g., rumination, brooding and worry) as related yet distinct constructs, we deemed it more conservative to measure trait PC (in the form of repetitive negative thinking and state PC (state rumination) via singular constructs as a measure of PC as a whole. This approach is supported by the notion that the most successful interventions of reducing PC, have also been noted to result in parallel, simultaneous, reductions to the main facets of PC (e.g., rumination and worry) [30].

## 2. Materials and Methods

### 2.1. Design

In this between subjects design, the Qualtrics randomizer element was employed to randomly allocate participants (at a ratio of 1:1) to either an experimental condition (where there was a 4:2 ratio in favour of lower calorie snacks) or control condition (in which there was a balanced ratio of 3:3 for both high and lower calorie snacks). Due to this device, neither the researchers nor participants knew which condition participants would be allocated. The two primary predictors under investigation in were: (a) trait-based PC (assessed via the PTQ) to evaluate the intrinsic, more stable, impact of PC on snack choice and (b) state-based PC (measured via the Brief State Rumination Inventory (BSRI)) to assess whether momentary PC motivates snack choice. Rumination, in particular, was selected as the state-based measure of PC because increases in rumination (*r* = 0.122), but not worry (*r* = 0.048) are associated with significantly greater health risk behaviours [26].

### 2.2. Participants

The sample size for the current pilot study was determined using the standard formula n = 100 + 50(i); where *i* is the number of independent variables in a logistical regression for the primary aim of the study [37]. This required a minimum of 250 complete datasets to be recruited. The inclusion criteria requested that eligible participants were free of a diagnosis of a relevant eating disorder, were UK residents (to ensure that all the snack options were recognizable to participants) and routinely purchased/consumed snack items (such as savoury snack foods and confectionary). Full details of the demographics of sample (across conditions) are available in Table 1.

### 2.3. Materials

#### 2.3.1. Snack Choice Task

**Figure 1:**
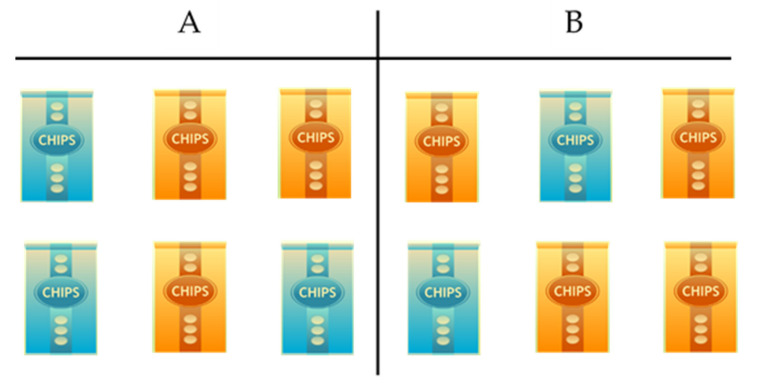
An illustrative example of the food choice task. Selection (**A**) demonstrates the even condition with a balanced distribution of ≥199 kcal (shown here in blue) and ≤99 kcal snacks (depicted in orange) (**B**) illustrates the uneven condition, whereby there was a 4:2 ratio in favour of the lower calorie snacks. Images are used for illustrative purposes only (adapted from: https://pixabay.com/illustrations/supermarket-shelf-products-snacks-1094815/ (accessed on 12 January 2021)).

Participants were presented with an array of 6 images of snack items (front of packet only) simultaneously on the screen, in a randomised order determined by Qualtrics. Items were displayed with the snack item name alongside the calorie content of the snack (in brackets). The participants were instructed to select the snack item from the array in which they would most like to consume “right now”. Participants completed this task twice, first with an array of crisps options and then again with an array of chocolate bars. Participants randomised to the even ratio condition for this task, would see a balanced ratio of 3 unhealthy items (defined as ≥199 kcal) and 3 healthier (defined as ≤99 kcal) for both the crisp and chocolate task. Equally, those randomised to the uneven ratio condition for the task saw the same array for both crisps and chocolate, with the exception that the ratio of healthier to unhealthy snacks was 4:2. All stimuli were presented in a randomised order. See an illustrative example in Figure 1 (below). Upon choosing their respective snacks for each task, participants were then asked to rate their “Enjoyment” of consuming the preferred snack on a 1 (“Not at all”)–7 (“Very much so”) Likert scale. A similar paradigm has been employed successfully in a previous investigation [6].

#### 2.3.2. Stimuli

All snack items included in the snack choice task were pre-packaged crisps and chocolate bars. These were chosen to reflect the preference of higher intake of foods high in fat and sugar, respectively, from individuals with higher stress levels. Thus, the decision of food choice under investigation here is that of choosing an option of lower calorie in contrast to higher calorie snacks. The healthier and unhealthy snacks were designed in line with the recommendations set out by Public Health England’s Change4Life recommendations. These propose that when an individual opts for a prepackaged snack, these should be 100 kcal or less, with the aim that these should be limited to two a day [38,39]. The current study allowed for the participant to make a selection of a high fat (in crisps) and high sugar (chocolate) snack while still being within the said endorsed recommendations. The final included stimuli for the unhealthy, higher calorie snacks were as follows. For the crisp task: McCoys Flame Grilled Steak Crisps (50 g), Pringles Original (40 g) and Doritos Cool Original (40 g). For the chocolate task: Snickers (48 g), Galaxy Smooth Milk Chocolate (42 g) and Mars (51 g). Regarding the stimuli for the healthier, lower calorie snacks, for the crisp task these were: Walkers Quavers (16 g), Walkers Wotsits (23 g) Walkers French Fries Salt and Vinegar (18 g) and Walkers Squares (22 g). Finally, for the chocolate task these were: Milkyway (21.5 g), Kinder Chocolate bar (12.5 g), Cadbury Dairy Milk “Little Bar” (18 g) and Cadbury Fudge bar (22 g). The above were chosen based on YouGov polling in which ≥96% of respondents recognized the brand and or product [40,41].

#### 2.3.3. Questionnaires

##### The Perseverative Thinking Questionnaire (PTQ)

A 15-item one-factor measure of trait repetitive negative thinking. Participants respond to statements about how they typically think about negative experiences or problems on a 5-point scale (0 “Never”–>4 “Almost always”) [42]. For example, item 7 states “*Thoughts come to my mind without me wanting them*” (see [42] for full 15 items). Scores for this questionnaire ranges from 0 to 60, with higher sum scores indicating higher levels of repetitive negative thinking. The scale has shown to have optimal internal consistencies (α = 0.95) and promising re-test reliability (*r* = 0.69) in both non-clinical and clinical participants [42]. The scale also asserts considerable convergent validity with other repetitive negative thinking and rumination scales including the Response Style Questionnaire (*r* = 0.72) and the Penn State Worry Questionnaire (*r* = 0.70).

##### The Brief State Rumination Inventory (BSRI)

An 8-item inventory of state rumination see [43] for scale. Participants are presented with 8 individual statements regarding the participant repetitive negative thinking at the time of answering. For instance, item 5 states “*Right now, I am rehashing in my mind recent things I’ve said or done*”. Participants are required respond to each item, via a 100 mm visual analogue scale (VAS) ranging from “Completely Disagree” (0) to “Completely Agree” (100). Scores for the current study were calculated as a sum score of all eight responses. The scale has shown to excellent internal consistency (α = 0.91).

##### Visual Analogue Scale (VAS)

The use of VAS can be deemed a quick and easy method of measuring human states. Two separate VAS were employed to measure self-reported stress and hunger. Participants were presented with the relevant statement (e.g., “How stressed are you”) where they were asked to indicate their response via a 100 mm line on the screen; describing the two extremes of the specific mood being measured (“Not at all”–“Extremely”), from left (0) to right (100). Participants were asked to indicate how hungry and stressed (respectively) they felt at that particular moment. These two VAS scales were presented after the snack choice specifically, to avoid highlighting or prompting the role of stress and appetite in snack choice to the participant.

### 2.4. Procedure

Participants were recruited by opportunity sampling via a number of social media posts and online survey share forums, between the months of November 2020 to January 2021. Upon following the online link, participants were presented with an information sheet fully outlining the details of the study and relevant inclusion criteria before they provided informed consent. Next, all eligible participants completed a series of demographic questions (full demographics can be found in Table 1), followed by the two snack choice tasks and after, the two VAS measuring self-reported hunger and stress, respectively. Participants finally completed the BSRI and PTQ before being presented with a debrief form. The survey took around 10 min to complete in full.

### 2.5. Ethics

The current study received ethical approval for the School of Education and Social Sciences at the University of the West of Scotland (Approval number: 13382; 11653).

### 2.6. Analysis

The current study was analysed via two logistic regressions, one for each snack study. The dependent variable was whether the participant chose the ≥199 kcal snack (coded 0) or the ≤99 kcal snack (coded 1) for the relevant crisp or chocolate paradigm. The primary predictors included in the main model were the condition (either even or uneven) and the total (sum) scores from the BSRI and PTQ, with control variables being gender and self-reported hunger. Data were analysed in SPSS (version 25), before being cross-validated in R-Studio (version 3.6.2) via two binomial logistic regression models (one per snack choice task). If state (BSRI) or trait (PTQ) PC scores were shown to influence snack choice, further analysis assessing the potential interaction between the relevant significant predictor and condition were planned. For ease of interpretation, results are reported in the form of odds ratio.

## 3. Results

### 3.1. Treatment of Data

The present study utilized complete-case-analysis, meaning incomplete data sets were first removed (N = 70) before the analysis was conducted. This was due to these cases not having the relevant include responses on the studies’ main outcome variables. One participant’s data was removed due to data catchment errors and one further dataset was removed due to the participant failing to meet the inclusion criteria. The final sample comprised 212 complete datasets for analysis. A summary of the final sample and their relevant demographics can be found below in Table 1. Given the skew in the data for both self-report hunger and scores from the BSRI, these were log (10) transformed prior to their inclusion into the regression model. Any interpretation of odds ratios for these continuous variables has been adapted accordingly.

### 3.2. Effect of PC on Snack Choice

The analysis revealed that both state and trait PC had a non-significant main effect on snack choice for both crisps (Hypothesis 1a) and chocolate (Hypothesis 1b). Higher levels of state PC were found to increase the likelihood of opting for one of the higher calorie crisp snacks (OR: 1.80; CI: 1.07–3.48), but this was only trending towards significance (*p* = 0.092). Given the absence of significant main effects of state and trait PC, no subsequent exploratory analysis was conducted regarding interactions between PC and the availability conditions.

### 3.3. Effect of Condition on Snack Choice

**Figure 2:**
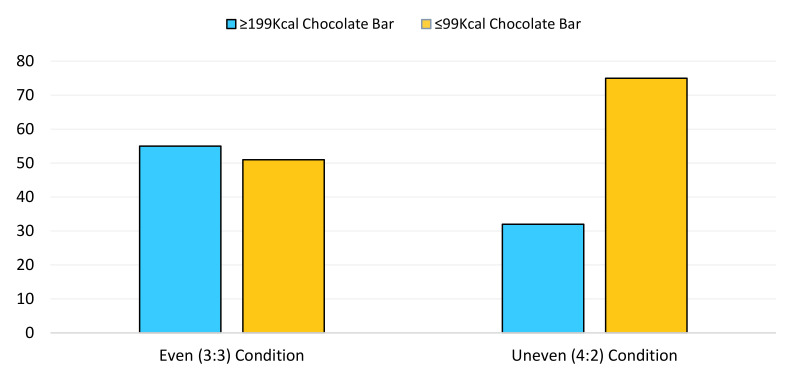
The number of 99 kcal (depicted in orange) and 199 kcal (presented in blue) chocolate bar selections made across the even (3:3 availability) and uneven conditions (4:2 availability in favour of the 99 kcals).

**Figure 3:**
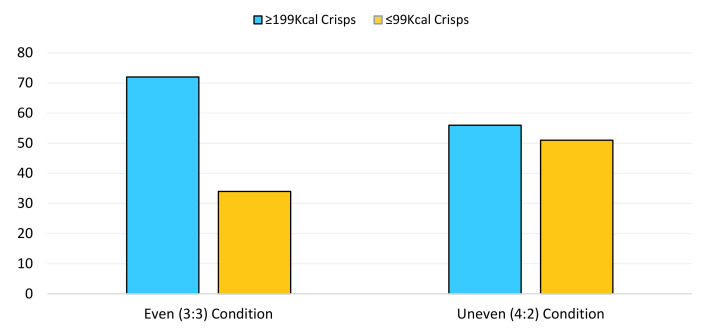
The number of 99 kcal (depicted in orange) and 199 kcal (presented in blue) crisp selections made across the even (3:3 availability) and uneven conditions (4:2 availability in favour of the 99 kcals).

The logistic regression models demonstrated a significant main effect of condition for both the crisp (*p* = 0.029; Hypothesis 2a) and chocolate (*p* = 0.002; Hypothesis 2b) selection tasks. It was revealed that the odds of choosing one of the lower ≤99 calorie chocolate snack when an individual was in the uneven (4:2 availability in favour ≤99 kcal snacks) condition was over twice as likely (OR: 2.50; CI: 1.39–4.48) relative to those in the even (3:3 availability) condition for the chocolate selection task (Figure 2). In addition, in the crisp snack selection task, those in the even (3:3 availability) condition were nearly twice as likely to choose the higher ≥199 calorie snack (OR: 1.93; CI: 1.07–3.48) relative those in the uneven (4:2 availability in favour ≤99 kcal snacks) condition (see Figure 3).

### 3.4. Covariates

The analysis showed that sex was a significant contributor to the final regression model for both the crisp (*p* = 0.014) and chocolate (*p* = 0.046) snack selections. It was found that females were nearly twice as likely to choose a lower calorie chocolate bar relative to males (OR: 1.85.; CI: 1.01–3.37), and males were more than twice as likely to choose the high calorie crisp option in contrast to females (OR: 2.23; CI: 1.17–4.24). Self-reported hunger was found to also be a significant covariate within the crisp tasks (*p* = 0.028), with higher levels of hunger increasing the likelihood of selection of higher calorie options (OR: 1.80; CI: 0.91–3.57). This finding was replicated in the chocolate selection task (OR: 1.69; CI: 1.05–2.70), but this was only found to be trending towards significance (*p* = 0.094). A summary of the final model for both logistical regression analyses can be found be in the Supplementary Materials (Tables S1 and S2).

## 4. Discussion

The current study investigated the role of PC in the selection of snacks (dense in sugar and fat, respectively) when in light of a varied availability of high and low calorie options of said snacks. PC has been hypothesized to exacerbate the relationship between stress and adverse health outcomes; including that of poorer dietary choices. The results of the current study showed, as predicted, that participants were significantly more likely to opt for the higher calorie crisp snacks when in the even condition (3:3 ratio of high and low kcal snacks) relative to the uneven condition (4:2 ratio in favour of lower kcal snacks). While, participants in the uneven condition (4:2 ratio in favour of low kcal snacks) were significantly more likely to opt for the lower calorie chocolate bar in contrast to those in the even condition (3:3 ratio of high and low kcal snacks). Regarding PC, higher levels of state rumination were found to increase the likelihood of the selection of higher calorie crisp snacks, but this was found to only be trending towards significance. Similarly, all other aspects of PC were not significantly related to snack choice for both chocolate and crisp snacks.

The of lack of significant influence from either trait PC or state rumination on snack choice is somewhat surprising. However, a number of explanations present themselves in order to explain these unanticipated findings. Firstly, the average scores for both trait PC and state rumination of the sample were modest, which may suggest that the participants, do not or were not engaging in repetitive negative thoughts to a level that is sensitive enough to be measured within the snack choice current paradigm. Additionally, and alternatively, the limited scheduling in which the PC measures were taken may not reflect the individuals’ true state and trait PC. As noted by Clancy and colleagues [44], not only are retrospective trait scales open to recall bias, but singular measurements of state scores do not always adequately capture the relative temporal fluctuations that naturally occur in these states. Consequently, the authors propose that the employment of multiple daily subjective (e.g., ecological momentary assessment) and objective measures of stress may assist in the unravelling of the interactions between, PC, stress and health behaviours [44].

A further explanation for the non-significant influence of PC could be due to levels of self-control acting as a potential mediator in PC related risk health behaviours, which was not measured here [45]. Indeed, several of the health risk behaviours associated with PC, such as binge drinking, substance abuse and binge eating [26,46,47,48] are also linked to a reported lack of self-control [48,49,50,51]. For example, individuals with higher levels of rumination demonstrated further symptoms of bulimia when they simultaneously reported inverse levels of self-control, even when controlling for the participants BMI [48]. While, other evidence has shown that rumination leads to uncontrolled eating, which may result in individuals seeking to modulate their own negative affect through food intake [52]. Therefore, it is imperative that future studies consider the impact of PC on known mediators of inhibition (such as self-control and executive functioning), supported through self-report, behavioural and physiological measures.

On a similar note, the current study measured one aspect of improving the quality of snack choice (i.e., lower kcal) rather than quantity. It is entirely possible that the volume of food consumed by those with high levels of PC is a more sensitive measure to explore further. As mentioned above, if PC is related more towards uncontrollable snacking then the employed paradigm, forcing participants to make a singular choice, would not have allowed any insight into whether individuals would have picked more than one option and in itself mitigated the role, or lack of, self-control in such choices. That being said, looking at whether this effect could be extended to single choice foods was still worthwhile. Indeed, while it may be argued that both snack choices presented in the proposed paradigm are palatable “comfort” foods, it is noteworthy that stress is associated with a preference for more calorie-dense options. Consequently, whether this preference is sensitive enough to be observed in singular snack choices was certainly worth exploring considering the ramifications for interventions and the public health goal of encouraging individuals to purchase lower calorie products [53].

Given the small sample size here and the trending influence of higher state PC on high calorie snack choice, it is important not to rule out that momentary rumination will lead to unhealthier, singular snack choices. Further work should logically seek to examine a similar project with a larger sample size. In addition, a reasonable extension of this work would be to investigate the theoretical behavioural and physiological underpinnings of PC-induced preferences for higher calorie snacks. For example, an attentional bias towards higher calorie dense foods has been found in overweight and obese populations relative to healthy weight controls see [54] for a review. It is interesting to consider whether such a bias may exist to partially explain the hypothesized preference for higher calorie foods in higher ruminating individuals, or, given the bi-directional nature of executive functioning and obesity [55], whether high rumination may exacerbate the existing bias in overweight and obese individuals. Additionally, and alternatively, in light of the suggestion that the consumption of comfort food may be used to temporally offset PC (particularly rumination [26]), such a coping mechanism may be orchestrated by poorer emotional regulation. Indeed, it is well established that PC leads to attentional and affective inflexibility, resulting in the consequential interruption of adaptive inhibitory processes [24,56]. While recent research has shown emotional dysregulation can negatively influence dietary choices in an attempt to self-regulate emotions [57], which may be impaired further by a higher BMI [58]. Thus, examining the mediating role of impaired emotional regulation, attentional bias and or a diminished inhibitory control in healthy and obese populations may help further understand the consumption of comfort food as a coping strategy in high ruminating individuals.

The results of reduced availability of unhealthy snack choices leading an individual to be less likely to choose a higher calorie snack (relative to a balanced availability) is in line with previous investigations [6,59] and adds to the growing literature of the potential benefit of using such behavioural economic tactics to improve acute dietary choices [4,5]. The results from this pilot investigation, would certainly suggest that such a snack choice paradigm may be a useful tool going forward to provide an insight to eating behaviours. Indeed, at the time of writing, many countries continue to impose heavy restrictions on the access to canteens, cafés and other eateries whereby individuals would usually make their daily choices, limiting the amount of ecological valid research in this area. However, while such online research paradigms would usually be deemed less ecologically valid, as grocery and takeaway food purchases shift from physical to digital [60], the ongoing restrictions may conversely afford this method to be a more valid research approach; resembling those of takeaway apps, for example. Future work should therefore seek to expand on the paradigm employed here in a larger sample and with a wider variety of snack choices, to ascertain which mechanisms (i.e., repeat exposure to a snack; snack familiarity; habitual response patterns; volume of snack availability) best explain dietary choices. In turn, this would cast new light on the most effective behavioural nudge tactics to influence snack choice; thus, arming prospective inventions with new tools to mitigate against the harmful impact of spontaneous unhealthy snacking to health.

Beyond those already discussed, the current pilot has further limitations. It is noteworthy, that a number of other social, economic, and biological explanations also influence appetite and food choice that have not been measured here [61,62,63]. Moreover, the fact that the current research has been carried out during a global pandemic may have had unexpected consequences on the data, particularly given the nature of the study. It may have been more prudent to also examine chronic stress levels and perhaps even self-reported changes to diet and physical activity because of any restrictions in place to better understand the lack of significant results.

## 5. Conclusions

The current study sought to examine the influence of state and trait PC when in light of a reduced availability of unhealthy snacks. Despite a trending effect of state PC (rumination) on the crisp selection task, neither measurements of PC were shown to have a significant influence on snack choice for either crisps or chocolate snacks. Although, a reduced availability of unhealthy, higher calorie snacks was found to significantly reduce the likelihood of choosing a higher calorie snack relative to an even availability of high and low calorie snacks. It is likely that the lack of contribution from PC is the result of the snack choice paradigm itself. The forced option paradigm may have inadvertently mitigated the contribution of PC-induced reductions to self-control in food choice. Further work should aim to investigate the influence of PC on food choice, while establishing the potential modulating role of other underlying cognitive (such as self-control) and affectual (e.g., emotional regulation) mechanisms. The ramifications of continued investigation into this area are vital for understanding stress-induced emotional eating and the subsequent identification of suitable cognitive and behavioural interventions that seek to attenuate this and the corresponding detrimental consequences to health.

## Figures and Tables

**Table 1:** Demographic information of the sample and across the two independent conditions. Due to data catchment errors and missing data points, only complete descriptive datasets are reported here. The corresponding N is reported for each demographic aspects.

		Even Condition (N = 105)	Uneven Condition (N = 107)	Overall (N = 212)
Age	N	105	106	211
	Mean	29.77	29.65	29.71
	SE mean	1.14	1.04	0.77
Sex	Females	66 (62.9%)	73 (68.2%)	139 (65.6%)
	Males	39 (37.1%)	34 (31.8%)	73 (34.4%)
BMI	N	92	103	195
	Mean	24.16	24.19	24.18
	SE mean	0.66	0.47	0.4
Stress	N	105	107	212
	Mean	51.5	42.45	46.93
	SE mean	2.82	2.79	2.08
Hunger	N	105	107	212
	Mean	40.57	36.42	38.48
	SE mean	2.82	2.71	1.96
State Rumination	N	105	107	212
	Mean	351.69	309.65	330.47
	SE mean	21.72	19.05	14.46
Trait Preservative Cognition	N	105	107	212
	Mean	34.4	31.32	32.84
	SE mean	1.22	1.14	0.84
Health Status	Poor	4 (3.8%)	1 (0.9%)	5 (2.4%)
	Fair	18 (17.1%)	10 (9.3%)	28 (13.2%)
	Good	34 (32.4%)	42 (39.3%)	76 (35.8%)
	Very Good	35 (33.3%)	37 (34.6%)	72 (34.0%)
	Excellent	14 (13.3%)	17 (15.9%)	31 (14.6%)
Weekly	≤14 Units a week	45 (42.9%)	43 (40.2%)	88 (41.5%)
Alcohol	≥15 Units a week	50 (47.6%)	56 (52.3%)	106 (50.0%)
Consumption	Do not drink	10 (9.5%)	8 (7.5%)	18 (8.5%)
Smoking Status	Non-smoker	71 (67.4%)	84 (78.5%)	155 (73.1%)
	Smoker	12 (11.5%)	10 (9.3%)	22 (10.4%)
	Occasional Smoker	2 (1.9%)	0 (0%)	2 (0.9%)
	Quitting (currently)	20 (19.0%)	13 (12.1%)	33 (15.6%)
Relationship Status	Single, never married	55 (52.4%)	65 (56%)	120 (56.6%)
	Married	30 (28.6%)	20 (18.7%)	50 (23.6%)
	Separated	2 (1.9%)	1 (0.9%)	3 (1.4%)
	Living with partner	18 (17.1%)	19 (17.8%)	37 (17.5%)
	Widowed/Divorced	0 (0.0%)	2 (1.9%)	2 (0.9%)
Household Income	£0–£14,000	30 (28.6%)	38 (35.5%)	68 (32.1%)
	£14,001–£24,000	21 (20.0%)	10 (9.3%)	31 (14.6%)
	£24,001–£30,000	13 (12.4%)	15 (14.0%)	28 (13.2%)
	£30,001–£40,000	9 (8.5%)	13 (12.1%)	22 (10.4%)
	£40,001–£80,000	22 (21.0%)	22 (20.6%)	44 (20.8%)
	£80,001+	10 (9.5%)	9 (8.4%)	19 (8.9%)

## Data Availability

The data presented in this study are available on request from the corresponding author.

## Supplementary Materials

The following are available online at https://www.mdpi.com/2076-328X/11/3/33/s1, Table S1: The model summary of the logistic regression predicting snack choice for the chocolate selection task. Primary predictors included were condition (availability ratio of high and low calorie snacks), state rumination and trait preservative cognition with gender and self-reported hunger incorporated entered as covariates. Table S2: The model summary of the logistic regression predicting snack choice for the crisp selection task. Primary predictors included were condition (availability ratio of high and low calorie snacks), state rumination and trait preservative cognition with gender and self-reported hunger incorporated entered as covariates.
