# Supplementary material for: Perseverative Cognition and Snack Choice: An Online Pilot Investigation
Source: Behav Sci (Basel). 2021 Mar 11;11(3):33. doi: 10.3390/bs11030033 (PMC7999187; doi:10.3390/bs11030033)
Supplement: Supplementary file 1 [file behavsci-11-00033-s001.pdf]

Table S1. The model summary of the logistic regression predicting snack choice for the chocolate selection task. Primary predictors included were condition (availability ratio of high & low calorie snacks), state rumination and trait preservative cognition with gender and self-reported hunger incorporated entered as covariates.

	Wald	*p*-Value	Odds Ratio	95% Conference Interval	
				Lower	*Upper*
**Male (Ref: Female)**	**3.992**	**0.046**	1.85	1.01	3.37
**Hunger**	2.805	0.094	1.69	1.05	2.70
**Uneven Condition (Ref: Even)**	9.436	0.002	2.50	1.39	4.48
**State Rumination (BSRI score)**	1.809	0.179	1.63	0.80	3.34
**Trait Preservative cognition (PTQ score)**	1.136	0.286	1.02	0.99	1.04
**Constant**	4.088	0.043	5.43		

Nagelkerke R Square = 0.129
Hosmer and Lemeshow Test: χ^2 = 6.065, p = 0.640

Table S2. The model summary of the logistic regression predicting snack choice for the crisp selection task. Primary predictors included were condition (availability ratio of high & low calorie snacks), state rumination and trait preservative cognition with gender and self-reported hunger incorporated entered as covariates.

	Wald	*p*-Value	Odds Ratio	95% Conference Interval	
				Lower	*Upper*
**Male (Ref: Female)**	**5.995**	**0.014**	2.23	1.17	4.24
**Hunger**	4.829	0.028	1.69	0.91	3.57
**Uneven Condition (Ref: Even)**	4.790	0.029	1.93	1.06	2.70
**State Rumination (BSRI score)**	2.834	0.092	1.80	1.07	3.48
**Trait Preservative cognition (PTQ score)**	0.059	0.808	1.00	0.98	1.03
**Constant**	2.601	0.107	3.65		

Nagelkerke R Square = 0.147
Hosmer and Lemeshow Test: χ^2 = 3.539, p = 0.896
